# Characterizing employment of colorectal cancer survivors using electronic health records

**DOI:** 10.1093/jamiaopen/ooab061

**Published:** 2021-08-02

**Authors:** Alexandra Varga, Inga Gruß, Debra P Ritzwoller, Cathy J Bradley, Andrew T Sterrett, Matthew P Banegas

**Affiliations:** 1 Kaiser Permanente Center for Health Research, Portland, Oregon, USA; 2 Institute for Health Research, Kaiser Permanente Colorado, Denver, Colorado, USA; 3 University of Colorado Denver, Colorado School of Public Health, Aurora, Colorado, USA

**Keywords:** electronic health record, employment status, occupation, cancer survivors

## Abstract

**Objective:**

Although the value of collecting occupational data is well-established, these data are not systematically collected in clinical practice. We assessed the availability of electronic health record (EHR)-based occupation data within a large integrated health care system to determine the feasibility of its use in research.

**Materials and Methods:**

We used a mixed-methods approach to extract EHR data and define employment status, employer, and employment industry of 1107 colorectal cancer survivors. This was a secondary analysis of a subset of the Patient Outcomes Research to Advance Learning (PORTAL) colorectal cancer cohort.

**Results:**

We categorized the employment industry for 46% of the cohort. Employment status was available for 58% of the cohort. The employer was missing for over 95% of the cohort.

**Conclusion:**

By combining data from structured and free-text EHR fields, we identified employment status and industry for approximately half of our sample. Findings demonstrate limitations of EHR data and underscore the need for systematic collection of occupation data in clinical practice.

LAY SUMMARY

Our study assessed whether employment information taken from an electronic health record (EHR) could be used in research. We examined the health records of 1107 colorectal cancer survivors receiving care from an integrated health system. We combined data from different parts of the health record, including insurance sources and free-text notes from providers. For the vast majority of patients, (95%), no specific employer was documented. However, we identified employment status for 58% and type of industry for 46% of this group. Our findings suggest that information about occupation and employment in the EHR is limited. To improve data collection, hospitals could integrate questions about patients’ occupation and employment into existing procedures using national standards.

## INTRODUCTION

Cancer treatment modalities have steadily improved over the last several decades, resulting in a growing number of individuals who are living as cancer survivors.^1^ By 2026, it is estimated that there will be over 20 million cancer survivors living in the United States.^2^ Thus, the American health system must help survivors manage the long-term medical and nonmedical effects of cancer and its treatment, including the long-term effects of cancer on employment.^3–5^ Cancer survivors often take time off from work or leave their jobs, and the process of re-entering the labor force can be complex, especially when the effects of cancer and its treatment persist.^6^ To address the employment needs of survivors, we need a better understanding of how cancer diagnosis affects employment trajectories, and how employment change affects long-term economic, socio-emotional, and health outcomes in this population. It is also important to better understand how occupational environments relate to health outcomes, including cancer progression and recurrence.

Systematically collecting and documenting occupational data in the electronic health record (EHR) can facilitate our understanding of the relationship between employment and health outcomes, facilitate care coordination, and help physicians and patients establish a return to work plan, ultimately improving care and population health.^7^^,^^8^ Although the value of collecting occupational data is well-established, data that contain information on employment status or occupation are not routinely or systematically collected by health care providers.^9^^,^^10^ In some health systems, EHR platforms include fields for employment status and occupation, but these fields are typically not mandatory for health care personnel to fill out. Previous studies have assessed costs and feasibility of translating and collecting occupational data, including through the development of coding systems have achieved mixed results.^11–14^ However, no previous research has examined EHR data and established how frequently employment and occupation fields are used or whether other EHR fields can be used to identify employment status and industry for research purposes.

In this study, a secondary analysis of a larger study using data from a subset of the Patient Outcomes Research to Advance Learning (PORTAL) colorectal cancer (CRC) cohort, we assessed the availability of EHR-based employment and occupation data within a large integrated health care system to determine the feasibility of use in research and clinical care. Our focus on CRC survivors was motivated by the rising incidence of CRC diagnosis in individuals who are of working age (25–64 years of age). Using a mixed-methods approach, we evaluated data from several Epic-based EHR fields,^1^ including free text fields, coded employment data, and occupation data for patients identified as survivors of CRC. We also developed a strategy for transforming this data into a usable research format to characterize employment status and occupation. Our assessment can inform future data collection and serve as a first step in investigating the relationship between employment and health and survivorship outcomes, which currently relies on primary data collection.

## METHODS

### Study setting

EHR data were collected from 2 Kaiser Permanente regions: Kaiser Permanente Northwest (KPNW) and Kaiser Permanente Colorado (KPCO). KPNW serves over 620 000 members in Oregon and Southwest Washington; KPCO serves over 630 000 members in communities in Colorado. Both regions were members of the PORTAL Network, funded by the Patient-Centered Outcomes Research Institute (PCORI), and are represented in the PORTAL CRC cohort, a cohort of over 16 000 patients from 6 health care systems diagnosed with CRC between 2010 and 2014,^15^ identified through medical records. The PORTAL CRC project aim was to use this cohort’s EHR data to gain insight into the lifestyles and health concerns of CRC survivors and to identify risk factors for health outcomes within this population. PORTAL cohort data were extracted using shared programming modules. Additional EHR data from these participants was later extracted from each site’s EHR. This study was approved by the KPNW Institutional Review Board.

### Population

Our study population comprised all PORTAL CRC cohort members who were members of KPNW and KPCO and met inclusion criteria (*n* = 1107). Members were included if they were between the ages of 18 and 70 at the time of a CRC diagnosis between January 1, 2010 and December 31, 2014, and were enrolled in the health plan at the time of diagnosis; those who had opted out of research participation were excluded. The data included both primary subscribers (insurance policyholders) and dependents, who were covered on the insurance of a primary subscriber.

### EHR data fields

We accessed EHR data from several sources. First, we retrieved PORTAL CRC cohort data from data sets that had been used in previous research and stored at each site, which included patient characteristics, tumor characteristics, and treatment details. We then extracted the additional employment and occupation data from each site’s EHR. These data included both patient-based data tables (tables that contain information about the patient such as demographics, marital status, etc.) based on data collected in clinical settings and insurance-based tables that contained information about patients’ insurance plans.

For all patients, we attempted to extract employer through a structured variable, *employer id*, on a patient-based table, and to link it to the employer name in a data table in the EHR that contains information on employers associated with patients and contracts with the health system. However, at one site, the employer id field was missing in 97% of the records. The other site had a combination of missing and a value of *Other* with no additional information in 95% of records. As data were available of less than 5% of the cohort, we opted not to further explore these data.

**Figure 1.**
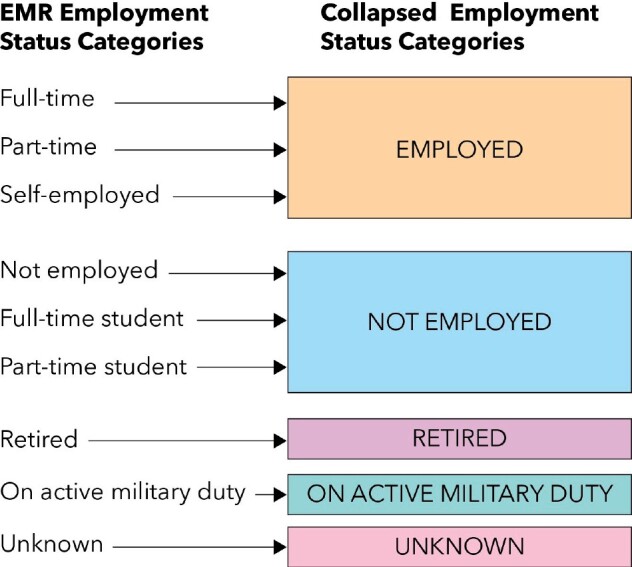
Flow diagram for categorization of employment status.

**Figure 2.**
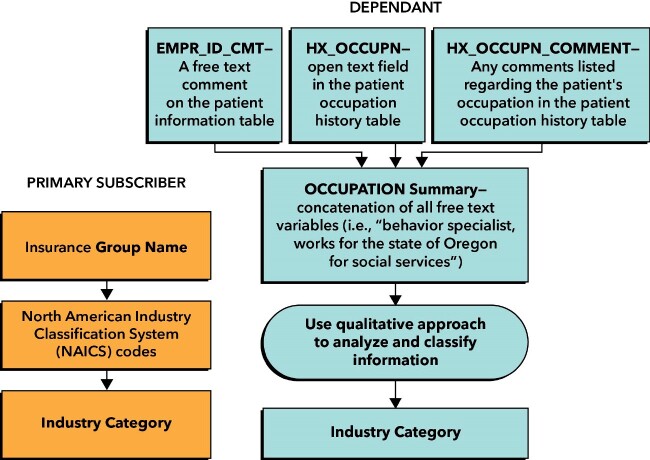
Flow diagram for categorization of employment industry.

To determine employment status and occupation for as many patients as possible, we examined structured employment status data and 3 open-text occupation fields from the patient-based tables (employer ID, occupation, and occupation comment), as well as health plan subscriber status and group. To health care providers, the structured employment and open-text employer and occupation fields appear in a pop-up box if a link is clicked in the demographics section at patient registration or check-in. These are not required fields for registration or check-in. Figures 1 and 2 provide an overview of how each of these data fields was used in our analysis.

#### Employment status

Employment status was available as a structured categorical variable in a patient-based table at both sites. This field contained the following options: full-time, part-time, self-employed, not employed, full-time student, part-time student, retired, on active military duty, and unknown. We collapsed the full-time, part-time, and self-employed categories into a single *Employed* category, and the not employed, full-time student, and part-time student categories into a single *Not Employed* category; the remaining 3 categories were unchanged (see Figure 1). The employment status categories are mutually exclusive; only one value may be endorsed.

#### Industry

For primary subscribers, we used insurance-based data to gather employment industry information. These data were not available for dependents, or for subscribers to state-funded or federally funded insurance programs, or for subscribers to Consolidated Omnibus Budget Reconciliation Act (COBRA) health insurance. For dependents, we extracted information about the employment industry from open-text EHR fields.

For *primary subscribers*, we obtained health plan group (ie, the employer or organization providing the health coverage) from insurance-based tables and linked this to its associated North American Industry Classification System (NAICS) code using the common membership group table (containing information for each insurance group contract) in the EHR. NAICS codes are standard codes, developed by federal agencies in North America (Office of Management and Budget), which are organized in a hierarchical structure for classification purposes. We categorized the NAICS codes into 20 economic sectors as defined by the United States Census Bureau; these sectors are defined using the first 2 digits of the NAICS codes. At KPCO, the insurance table included Standard Industrial Classification (SIC) codes (the predecessor of NAICS codes), which we converted to NAICS codes using a crosswalk from the US census website.^16^^,^^17^

For *dependents* of primary subscribers, we used 3 open-text EHR fields with information on employment and occupation history to classify the employment industry. The first field, “occupation history,” prompts providers to list a patient’s specific occupation. The second field is for any comments regarding the patient’s occupation. The third field is a free-text comment field that can be used to specify an employer when the value of the *employer ID* field is set to “Other.” These fields included a variety of information and comments about occupation (eg, truck driver, professor, waitress), employment status (eg, retired, unemployed, self-employed), type of work, industry, employer (eg, “US treasury,” “Warner Pacific”), and length of employment. Each field allowed for up to 250 characters (approximately 40–60 words). For each record, we concatenated all available free text into a single variable. The resulting variable ranged from 1 to 22 words; however, most records had fewer than 5 words. A qualitative analyst manually assessed and categorized the open-text data using the 20 NAICS-based industry sectors described above. If employer names were listed, the analyst used publicly available information to categorize the organization according to the NAICS-based industry sectors. While these fields were populated for some of the primary subscribers, we opted to maintain consistency by defining employment based on the health plan group for all primary subscribers. Figure 2 presents a graphical representation of the data flow.

### Evaluating feasibility of using EHR data for research

We assessed the number and proportion of primary subscribers and dependents whose employment status and industry category could be determined using the methods described above. For dependents of primary subscribers, we also determined the number of patients who had information in the occupation open-text field for whom we were not able to assign an industry category. We compared rates of data capture between the methods used for subscribers and dependents.

## RESULTS

### Sample characteristics

Characteristics of the 1107 CRC survivors in our study sample are presented in Table 1. 78.3% of survivors were primary subscribers (*N* = 867) and 21.7% were dependents (*N* = 240). There were 356 (32.2%) survivors over the age of 64: 309 primary subscribers (35.6%) and 47 dependents (19.6%). More than three-fourths of both primary subscribers and dependents were identified as non-Hispanic White. Almost all (90.8%) of the group received surgery for their cancer. Almost half of the group underwent chemotherapy (43.7%) and 15.4% underwent radiation therapy.

**Table 1.** Characteristics by subscriber status

	Dependent		Primary subscriber		All	
	(*N* = 240)		(*N* = 867)		(*N* = 1107)	
	*N*	(%)	*N*	(%)	*N*	(%)
Age, mean (SD)	56.62	(8.1)	59.29	(8.6)	58.71	(8.5)
Age						
18–39	9	(3.8)	22	(2.5)	31	(2.8)
40–54	86	(35.8)	221	(25.5)	307	(27.7)
55–64	98	(40.8)	315	(36.3)	413	(37.3)
65–70	47	(19.6)	309	(35.6)	356	(32.2)
Female	120	(50.0)	408	(47.1)	528	(47.7)
Race						
Asian	8	(3.3)	20	(2.3)	28	(2.5)
Black	7	(2.9)	33	(3.8)	40	(3.6)
Mixed race/other	5	(2.1)	24	(2.8)	29	(2.6)
Hispanic	17	(7.1)	80	(9.2)	97	(8.8)
Unknown	7	(2.9)	32	(3.7)	39	(3.5)
Non-Hispanic White	196	(81.7)	678	(78.2)	874	(79.0)
Insurance						
Commercial	177	(73.75)	476	(54.90)	653	(58.99)
Medicaid/Medicare	0	(0)	8	(0.92)	8	(0.72)
Medicaid	2	(0.83)	21	(2.42)	23	(2.08)
Medicare	55	(22.92)	326	(37.60)	381	(34.42)
CRC treatment^a^						
Surgery	220	(91.7)	785	(90.5)	1005	(90.8)
Radiation	29	(12.1)	141	(16.3)	170	(15.4)
Chemotherapy	109	(45.4)	375	(43.3)	484	(43.7)
Hormone treatment	2	(0.8)	4	(0.5)	6	(0.5)
Immunotherapy	5	(2.1)	19	(2.2)	24	(2.2)

^a^ Treatment categories are not mutually exclusive; a patient may have multiple therapies.

### Employment status

Table 2 reports employment status and industry category for all participants and for the subgroups of primary subscribers and dependents. Overall, 58% of patients in the sample (55.8% of primary subscribers and 65.8% of dependents) had an employment status entered in the structured EHR field. Of those, 53% were listed as employed, 4% as unemployed, 35% as retired, and 3% as unknown.

**Table 2.** Employment status and employment industry by subscriber status

	Dependent		Primary subscriber		All	
	(*N* = 240)		(*N* = 867)		(*N* = 1107)	
	*N*	(%)	*N*	(%)	*N*	(%)
Employment status						
Missing	82	(34.2)	383	(44.2)	465	(42.0)
Employed	82	(34.2)	257	(29.6)	339	(30.6)
Not employed	30	(12.5)	28	(3.2)	58	(5.2)
Retired	37	(15.4)	186	(21.5)	223	(20.1)
Unknown	9	(3.8)	13	(1.5)	22	(2.0)
Industry category						
Missing	166	(69.2)	432	(49.8)	598	(54.0)
Accommodation and food services	4	(1.7)	2	(0.2)	6	(0.5)
Administrative and support and waste management services	2	(0.8)	9	(1.0)	11	(1.0)
Agriculture, forestry, fishing, and hunting	1	(0.4)	1	(0.1)	2	(0.2)
Arts, entertainment, and recreation	2	(0.8)	4	(0.5)	6	(0.5)
Construction	7	(2.9)	4	(0.5)	11	(1.0)
Educational services	9	(3.8)	43	(5.0)	52	(4.7)
Finance and insurance	3	(1.3)	53	(6.1)	56	(5.1)
Health care and social assistance	7	(2.9)	31	(3.6)	38	(3.4)
Information	0	(0.0)	6	(0.7)	6	(0.5)
Management of companies and enterprises	1	(0.4)	0	(0.0)	1	(0.1)
Manufacturing	5	(2.1)	41	(4.7)	46	(4.2)
Other services, except public administration	6	(2.5)	44	(5.1)	50	(4.5)
Professional, scientific, and technical services	4	(1.7)	16	(1.8)	20	(1.8)
Public administration	7	(2.9)	125	(14.4)	132	(11.9)
Real estate and rental and leasing	0	(0.0)	6	(0.7)	6	(0.5)
Retail trade	6	(2.5)	32	(3.7)	38	(3.4)
Transportation and warehousing	4	(1.7)	12	(1.4)	16	(1.4)
Utilities	4	(1.7)	2	(0.2)	6	(0.5)
Wholesale trade	2	(0.8)	4	(0.5)	6	(0.5)

### Industry

Using NAICS codes from insurance-based tables, we identified the occupational industry for 435 (50.2%) of primary subscribers. The most commonly identified industries for subscribers were public administration (14.4%), finance and insurance (6.1%), other services except public administration (4.5%), and educational services (5.0%).

Employment data were available in one or more of the 3 free-text fields for 113 (47.1%) of dependents; however, we were only able to categorize 74 of those records (65%) into the 20 occupational categories, resulting in industry data for 30.8% of the total dependent sample. The most common industries for dependents were educational services (3.8%) and construction, health care and social assistance, and public administration (each accounting for approximately 3% of the dependent sample).

### Availability of data sources

**Figure 3.**
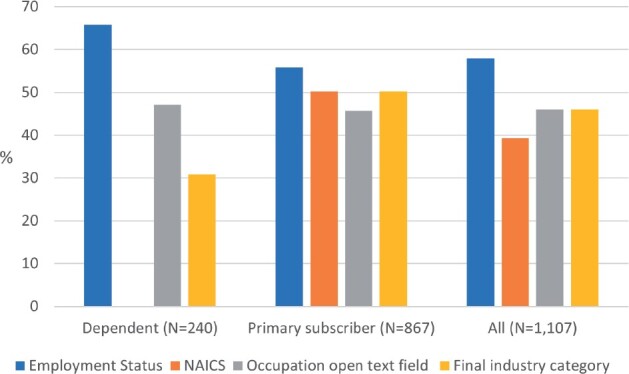
Employment data availability by subscriber status.

Figure 3 reports the availability of each data source across subscribers and dependents. Across all patients in the cohort, 58% had a value in the employment status field, and 39.3% (50.2% of subscribers; NAICS were relevant for subscribers only) had NAICS codes in insurance-based tables. Overall, 46% of patients had information in an occupational text field; these rates were comparable between primary subscribers (45.7%) and dependents (47.1%). Our analysis of data for the sample of dependent subscribers suggests that information from these free-text fields was sufficient to identify an industry category in 65.5% of the records that had free-text data. Thus, using analysis of the free-text fields, we identified the industry for 30.8% of dependents, and using NAICS codes from the insurance-based table, we identified industry for 50.2% of subscribers. Taken together, we identified industry category for 46% of patients in the cohort.

## DISCUSSION

Using a comprehensive data capture method that combined input from structured and free-text EHR fields with subscriber data across 2 health systems, we captured the employment status of 58% of the CRC survivors in our sample. We were also able to capture the employment industry for 46% of survivors in the sample. While we had a priori decided to only categorize free text for the dependent sample, there was free-text data available on 201 of the primary subscribers who did not have NAICS data. Assuming the same rate as the dependents (65%), we would have been able to classify approximately 130 more primary subscribers who had no other employment data available, bringing the total up to 639 or about 58% of all patients. Even with the possibility of categorizing additional patients, these findings suggest that EHR data may be useful as a foundation or supplement for primary data collection approaches used in past research on employment in cancer survivors,^18–21^ but not as the principal or only source for such data. EHR data may serve as a useful supplementary source of data to enhance occupational data capture, although we note that the high rates of missing data limit the utility of these data source and highlight the potential research benefit of standardized collection of employment data in health care settings. Having more rigorous surveillance data would enable us to identify risk factors (eg, occupation) on which we can plan in-depth research through more enhanced methods.

To improve data collection about occupation and employment, hospitals could integrate more structured questions about occupation and employment with clear answer options into existing hospital procedures, making use of current federal coding practices (NAICS and SOC codes), as recommended by the Institute of Medicine^8^ to do so. Health systems have implemented routine screening practices for other social determinants of health, including housing stability and food security, sugary beverage intake, and physical activity levels, without impeding clinical flow.^22–24^ This suggests that collecting occupation and industry information should also be possible, especially for populations (such as cancer survivors) for whom these data may be especially valuable for both clinical and research purposes. Collecting this information at intake would allow for temporal associations, including tracking changes in employment over time and association with diagnosis date, treatment, and other clinical factors.

Researchers have suggested several solutions to the problem of accessing occupation data that is recorded in free-text fields, including standardized coding systems and manual administrative coding,^11^ although neither have been fully implemented that we are aware of. Manual administrative coding of patient responses to employment-related questions is labor intensive for large amounts of data. The National Institute for Occupational Safety and Health (NIOSH) has developed a coding algorithm, the NIOSH Industry and Occupation Computerized Coding System (NIOCCS) that coverts free text into SOC and NAICS codes (NIOSH/CDC website). The system has potential as an accurate and efficient strategy for reducing expenditure of valuable time and money in the health delivery system.^9^ However, field tests have demonstrated lower accuracy than NIOSH monitoring rates, and insufficient analyzable data for some records, indicating that improving the collection methods of employment and industry data, such as more targeted questions or better training of administration staff, could ameliorate the usability of the NIOCCS software.^11^^,^^13^ Importantly, in our sample, data from free-text fields only contained valuable information for 30% of dependent patients. This suggests that beyond identifying easy ways to code these data in standardized formats, efforts need to be made to increase data collection (less than half of the patients had information in any of the 3 free-text fields in the EHR) and to ensure the occupation is captured in data collection (nearly 35% of dependents with free-text fields could not be categorized by our expert qualitative researcher) for these data to be a reliable source of industry of employment. Additional text mining methods such as natural language processing may reveal additional information about the occupational status.

Our study confirms what previous research has suggested: routine collection and documentation of occupational data in the EHR are necessary to make these data useful for research and ultimately clinical outcomes. Attempts have been made to establish federal requirements for collecting employment data, including a petition the National Uniform Billing Committee (NUBC) to adopt industry and occupation coding standards to hospital discharge data, however, the petition was ultimately rejected due to concerns about undue burden on administration and health care providers.^9^^,^^14^ Follow-up studies have found that this concern may be unfounded, as it was possible during pilot studies to collect and document these data at a low cost and without impeding clinic workflows.^12^^,^^14^ Such information could also be collected through EHR-based questionnaires or flowsheets.

There were several limitations to our study. We assessed the feasibility of this approach at 2 sites within one health care system only, and only among CRC survivors. Focusing on CRC survivors enabled us to review the data in detail, but may limit the generalizability of our findings. Our findings may also not be generalizable to other health care systems. Members who are primary subscribers and get their insurance through their employer are overrepresented in the data for the occupational industry; also, identifying industry may have been easier for large, centralized employers, leading to higher rates of identification in industries like public administration. Lastly, there was no validation process to ensure that the data that were recorded in the EHR were accurate according to the patient.

## CONCLUSION

Using different types of data from multiple sources, in addition to qualitative analysis techniques, we were able to combine insurance subscriber data with data garnered from structured and free-text EHR fields to identify employment status and industry for approximately half of the CRC survivors in our sample. There is growing interest in documenting social determinants of health (SDoH) in the EHR.^25^ Employment and occupation are important factors of individual SDoH. However, SDoH are not often well documented in the EHR,^26^ which is underscored by our findings.

These results demonstrate the strengths and limitations of currently available occupation data in the EHR, and underscore the need for routine and precise collection of such data in clinical practice. Better collection of employment and occupational data would facilitate critical research and quality improvement efforts focused on the interplay between occupation, employment, and health. Along with other SDoH data, improving the documentation of occupation and employment is important for improving individual- and population-level health care through a better understanding of patients’ economic circumstances and health exposures. As the Institute of Medicine recommends, adopting NAICS coding standards and exploring the feasibility of autocoding are important steps to better document these important elements of SDoH.^8^

## FUNDING

This study used the infrastructure developed by the PORTAL (Patient Outcomes Research to Advance Learning) Network, a consortium of 3 integrated delivery systems (Kaiser Permanente, HealthPartners, and Denver Health) and their affiliated research centers. Research reported in this article was partially funded through a Patient‐Centered Outcomes Research Institute (PCORI) Award (CDRN‐1306‐04681 Phase II). The views expressed in this article are solely the responsibility of the authors and do not necessarily represent the views of the Patient-Centered Outcomes Research Institute (PCORI), its Board of Governors, or Methodology Committee.

## AUTHOR CONTRIBUTIONS

AV, IG, and MPB had substantial contributions to the conception and design of the work. MPB and DPR were responsible for data acquisition. Analyses were lead and/or conducted by AV, IG, MPB, and ATS. Interpretation of data for the work was done by AV, IG, MPB, CJB, DPR, and ATS. AV, IG, MPB, CJB, DPR, and ATS contributed intellectual contact in the drafting and revision of the manuscript. All authors approved of the final version.

## DATA SHARING

The data used in this study cannot be shared publicly due to privacy concerns for and agreements with Kaiser Permanente members.

## ETHICAL CONSIDERATIONS AND DISCLOSURE(S)

The study was performed in accordance with the ethical standards of the institutional and/or national research committee and with the World Medical Association Declaration of Helsinki (1964) and its later amendments or comparable ethical standards. The study was approved by the Institutional Review Board at Kaiser Permanente Northwest.

## Footnotes

1 Epic is a provider of health information technology including an EHR software application system.
